# Protein-Phospholipid Interactions in Nonclassical Protein Secretion: Problem and Methods of Study

**DOI:** 10.3390/ijms14023734

**Published:** 2013-02-08

**Authors:** Igor Prudovsky, Thallapuranam Krishnaswamy Suresh Kumar, Sarah Sterling, David Neivandt

**Affiliations:** 1 Maine Medical Center Research Institute, 81 Research Drive, Scarborough, ME 04074, USA; 2 Department of Chemistry and Biochemistry, University of Arkansas, Fayetteville, AR 72701, USA; E-Mail: sthalla@uark.edu; 3 Department of Chemical and Biological Engineering, University of Maine, Orono, ME 04469, USA; E-Mails: sarah.sterling@maine.edu (S.S.); dneivandt@umche.maine.edu (D.N.)

**Keywords:** FGF, phospholipid, nonclassical secretion, S100A13, sphingosine kinase 1, annexin 2, phospholipid, SFS, FCS, liposome, supported bilayer, copper, synaptotagmin 1

## Abstract

Extracellular proteins devoid of signal peptides use nonclassical secretion mechanisms for their export. These mechanisms are independent of the endoplasmic reticulum and Golgi. Some nonclassically released proteins, particularly fibroblast growth factors (FGF) 1 and 2, are exported as a result of their direct translocation through the cell membrane. This process requires specific interactions of released proteins with membrane phospholipids. In this review written by a cell biologist, a structural biologist and two membrane engineers, we discuss the following subjects: (i) Phenomenon of nonclassical protein release and its biological significance; (ii) Composition of the FGF1 multiprotein release complex (MRC); (iii) The relationship between FGF1 export and acidic phospholipid externalization; (iv) Interactions of FGF1 MRC components with acidic phospholipids; (v) Methods to study the transmembrane translocation of proteins; (vi) Membrane models to study nonclassical protein release.

## 1. Introduction

The classic protein secretion mechanism, which involves endoplasmic reticulum (ER) and Golgi apparatus, enables the release of most extracellular proteins. All these macromolecules have, in their primary structure, a cleavable hydrophobic signal peptide, which usually locates *N*-terminally and is required for translocation of the nascent protein into the ER lumen [1]. However, many extracellular proteins lack signal peptides but still are successfully exported. Their release does not require ER-Golgi as demonstrated by its insensitivity to brefeldin, a specific inhibitor of protein transport from ER to Golgi [2,3]. Among the nonclassically secreted proteins are growth factors FGF1 [4] and FGF2 [5,6], pro-inflammatory cytokines IL1β [7,8], IL1α [9,10], IL33 [11], HMBG1[12,13], EMAP II [14] and MIF [15], enzymes sphingosine kinase 1 (SphK1) [16] and secretory transgultaminase [17], annexins 1 [18] and 2 [19,20], galectins [21–23], S100 family proteins [24–26] and others. Interestingly, some of these proteins, such as FGF1 and FGF2, execute not only extracellular but also intracellular functions [27,28], which could be hampered if the newly synthesized proteins were immediately directed to ER-Golgi. Many nonclassically exported proteins can exhibit harmful biological effects. For example, even very low levels of IL1β and IL1α in the bloodstream induce strong fever [29], while increased levels of FGF1 correlate with hereditary hypertension [30]. Apparently, the inability of signal peptide-less exported proteins to be released through the classical secretion pathway protects the organism from their undesirable effects. Consequently, the nonclassical protein export is tightly regulated and usually induced by specific stimuli, such as various forms of cell stress. Interestingly, *Caenorhabditis elegans* and *Drosophila melanogaster*, have only one form of FGF, which has a signal peptide [31]. Unlike round worms and insects, vertebrate have many FGF genes—twenty three in mammals [32], most of which are secreted proteins that act through specific cell surface receptors (FGFR). Unlike FGF1 and FGF2, which are ubiquitously expressed in the organism [33], other secreted FGFs have signal peptides and their expression is limited to certain tissues or cell types [32]. We hypothesized that the evolutionary loss of signal peptides in FGF1 and FGF2 protects the organism from the harmful effects of these potent regulators expressed in most tissues of the organism [2]. Nonclassical release of signal peptide-less FGFs under stress conditions that exist in damaged and inflamed tissue stimulates tissue repair, which in turn results in the cessation of stress and thus suppresses FGF secretion.

The key problem for any mechanism of protein secretion is how to overcome the hydrophobic membrane barrier. Some signal peptide-less proteins including IL1β can apparently be incorporated into autophagosomes and then released in exosomes [34,35]. Other nonclassically secreted proteins use unknown mechanisms to translocate from cytosol into the lumen of lysosome-like vesicular structures [8,12,36]. Both autophagosomes and lysosomes can fuse with the cell membrane and release their content to the extracellular compartment. However, there exist also signal peptide-less proteins, which directly translocate through the cell membrane. Among them are FGF2, FGF1 and mature IL1α [10,37,38]. These proteins do not show a dot-like vesicular localization in the cytoplasm. Instead, they are diffusely distributed in the cells, usually both in nuclei and cytoplasm. The released FGF1 and FGF2, which strongly bind heparan sulfate proteoglycans, are associated with the extracellular matrix [39,40]. In this review, we will focus on FGF1 export to discuss the structural aspects of nonclassical protein export, particularly protein-lipid interactions, and approaches to their study.

## 2. Molecular Determinants of the Nonclassical Export of FGF1

FGF1 is expressed in most tissues of the vertebrate organism [41]. Recombinant FGF1 has been used to accelerate the repair of wounds and post-ischemic tissues [42]. At the same time, natural or artificial FGF1 overexpression has been associated with tumorigenesis [33], hereditary hypertension [30], and fibrosis [43]. The nonclassical export of FGF1 was extensively studied using mouse fibroblasts [2]. Whereas under normal conditions FGF1 release is barely detectable, different forms of cell stress, such as heat shock [4], hypoxia [44], serum starvation [44] and cell treatment with oxidized lipoproteins [45] stimulate FGF1 export. Interestingly, FGF1 export can also be induced by the inhibition of Notch signaling [46,47] and by thrombin treatment [48,49]. Similar to cell stress, the decrease of Notch signaling (due to loss of cell-cell contacts) and the proteolytic activation of thrombin occur in damaged and ischemic tissues. In addition, the core of solid tumors is often characterized by hypoxic stress and enhanced expression of PAR1, the major receptor of thrombin [50]. Thus, wounding, ischemia and tumorigenesis involve local conditions propitious for FGF1 secretion.

**Figure 1:**
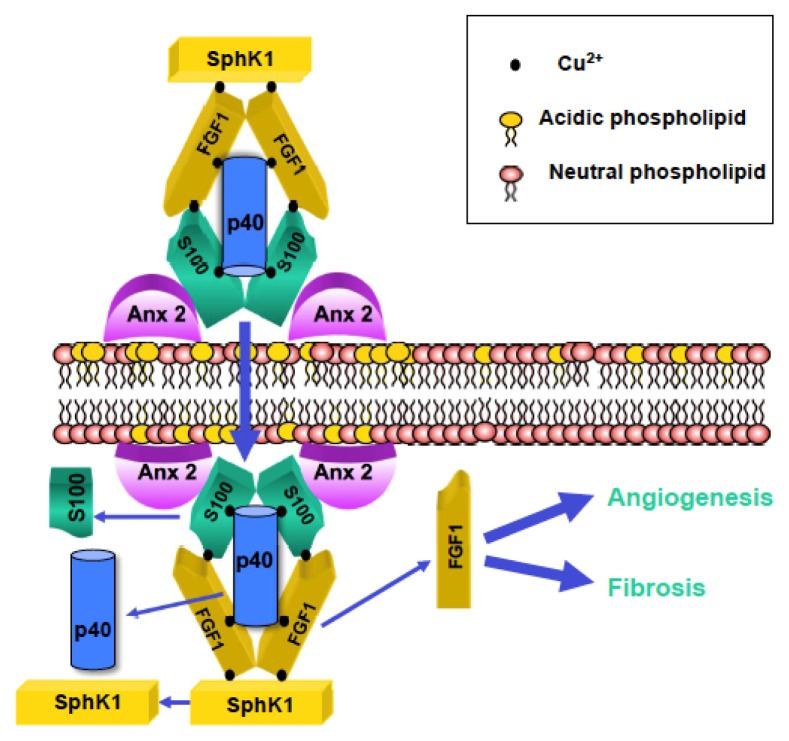
FGF1 multiprotein release complex (MRC): a hypothetical scheme. The copper-dependent MRC comprises a dimer of FGF1, sphingosine kinase 1 (SphK1), p40 synaptotagmin 1, S100A13 and annexin 2 (Anx 2). The assembly of MRC takes place at the inner plasma membrane leaflet of stressed cells. The externalization of MRC components depends on transmembrane translocation of acidic phospholipids.

At the molecular level, stress-induced FGF1 export is one of the best-studied cases of nonclassical protein secretion. FGF1 secretion depends on several other signal-peptide-less secreted proteins, such as the small calcium-binding protein S100A13 [24], the 40 kDa form of synaptotagmin 1 (p40 Syt1) [51] and the enzyme SphK1 [52]. These proteins together with a covalent (Cys30 disulphide linked) FGF1 dimer [53] comprise a copper-dependent [54] multiprotein release complex (MRC) (Figure 1). All MRC components have significant or, as in case of SphK1, high affinity to Cu^2+^ [52,54].

Copper chelation inhibits FGF1 export under stress conditions [52,54]. The experiments with dominant negative mutants [24,51] or genetic knockouts [52] and knockdowns [55] demonstrated the critical importance of FGF1 MRC proteins for the stress-induced FGF1 release. We hypothesized [2] that during the stress FGF1 MRC interacts with the signal peptide-less protein annexin 2 (Anx 2), which exhibits the stress-stimulated translocation through the cell membrane [19]. Anx 2 typically forms a heterotetramer composed of two Anx 2 and two S100A10 molecules [56]. Based on structural similarity between S100A13 and S100A10 [57] we suggest that S100A13 is responsible for the interaction between FGF1 MRC and Anx 2. The exact functions of FGF1 MRC components during the stress-dependent FGF1 secretion are not determined. Interestingly, S100A13, p40 Syt1 and SphK1 are released from the cells spontaneously, in the absence of stress. However, when they are co-expressed with FGF1, their export becomes stress-dependent [24,51,52]. The importance of FGF1 MRC for thrombin-induced and Notch-regulated FGF1 release is not sufficiently understood. However, SphK1 knockout inhibits the export of FGF1 induced by the suppression of Notch signaling [47].

## 3. Role of Cu^2+^ in the Organization of the FGF1 MRC

Cu^2+^ is mandatory for the non-classical secretion of FGF1 into the extracellular compartment [54]. However, the exact role of Cu^2+^ in the organization of the MRC of FGF1 is still not clear [58]. In addition, several other crucial questions in the Cu^2+^-mediated non-classical release of FGF1 remain unanswered. For example, what is the mechanism by which Cu^2+^ is recruited for the formation of the MRC? It is obvious that free Cu^2+^, due to its highly reactive and toxic nature, does not accumulate in the cell [59,60]. To circumvent this problem, cells have plausibly evolved a mechanism wherein Cu^2+^ ions necessary for the formation of the FGF1 MRC are provided by small copper-binding proteins called the “copper chaperones” [61]. These “copper chaperones” receive Cu^2+^ from cell-surface copper transporters and distribute them to the destination proteins that require Cu^2+^ for their function(s) [61,62]. Atx1, CCS, and Cox17 are some of the well-studied “copper chaperones” found in the cytoplasm of cells [63–65]. Although there is no experimental evidence of any of the “copper chaperones” interacting with the protein components in the multi-protein FGF1 release complex, it is reasonable to predict that the Cu^2+^ ions required for the formation of the FGF1 dimer are transferred from one of the “copper chaperones” to proteins involved in the FGF release complex. Another potential donor of Cu^2+^ required for FGF1 MRC formation is SphK1. Indeed, this protein has a very high copper affinity. While FGF1 is eluted from the copper column by 40–60 mM imidazole, SphK1 elution requires 500 mM imidazole [52]. Overexpression of SphK1 rescues FGF1 export from inhibition by copper chelator tetrathiomolybdate [52].

**Figure 2:**
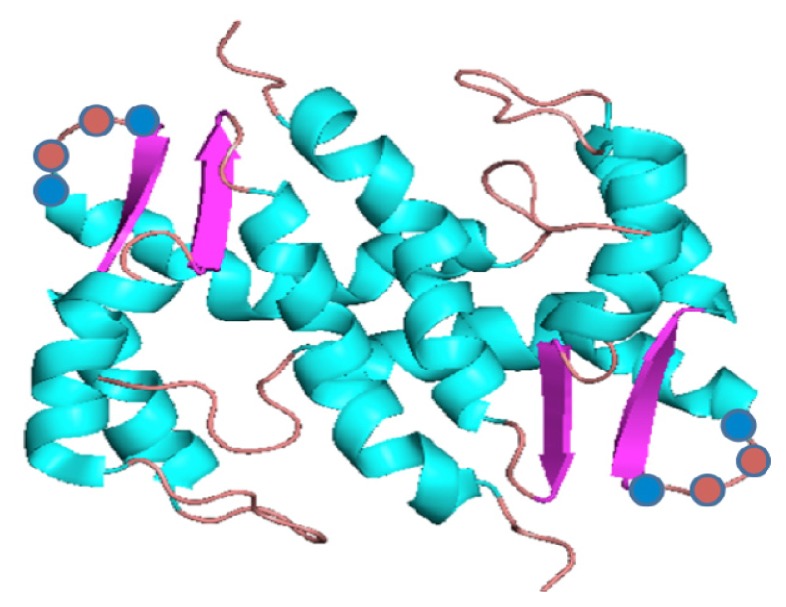
Three-dimensional structure of S100A13 with the bound Cu^2+^ (blue) and Ca^2+^ (red brick) ions.

Isothermal titration calorimetry (ITC) results suggest that S100A13 binds to Cu^2+^ with moderate affinity (*K*_d_ ≈ 12 μM) and a protein to Cu^2+^ binding ratio of 1:4 [66–68]. The binding affinity and binding stoichiometry of S100A13 to Cu^2+^ and Ca^2+^ are similar. Interestingly, the Ca^2+^ and Cu^2+^ binding to S100A13 are not mutually exclusive and protein can bind to both the metal ions simultaneously [68]. Binding of Cu^2+^ induces a subtle conformational change in S100A13. Nuclear magnetic resonance (NMR) data show that the Cu^2+^ and Ca^2+^ binding sites in S100A13 are topologically close but quite distinct [68]. The Cu^2+^ binding sites on S100A13 are distributed in two pockets. One site comprises of Leu33, Asn34, Leu71, and Arg72 located on the β-sheet between helix-3 and helix-4. The second Cu^2+^ binding site includes Thr7, Glu8, and His48, which are located in the hinge region between helix-2 and helix-3 (Figure 2).

**Figure 3:**
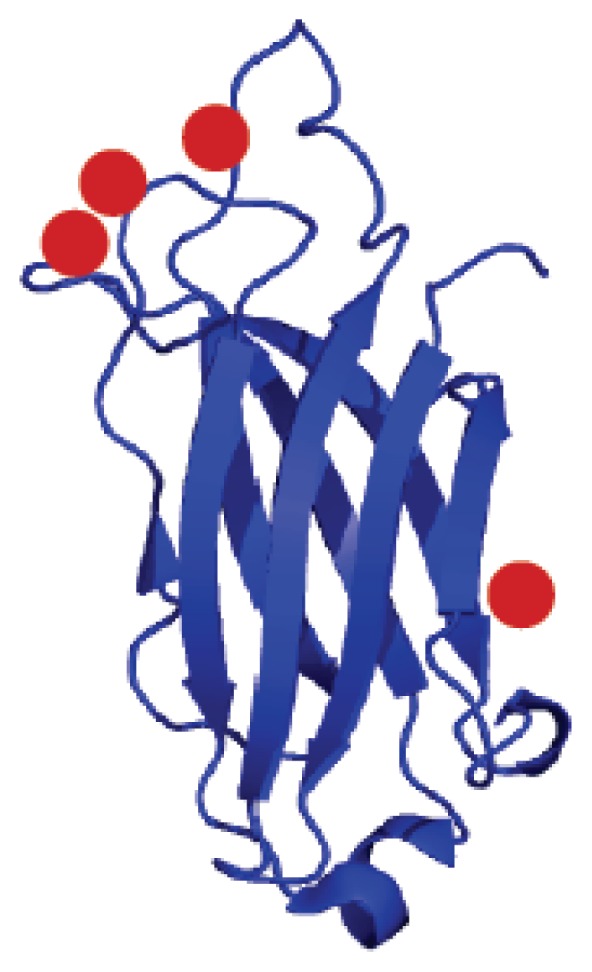
Three-dimensional structure of the complex between synaptotagmin 1 C2A domain and copper ions. Cu^2+^ ions are shown as red circles.

Synaptotagmins constitute a family of vesicle membrane proteins that are characterized by a short intravesicular *N*-terminus, a single transmembrane region, and a larger cytoplasmic region that contains calcium binding C2 domains, designated as C2A and C2B [69–71]. p40 Syt1 is a shorter form of Syt1, which is produced as a result of the alternative initiation of Syt1 mRNA translation [72]. Unlike transmembrane p65 Syt1, a protein involved in classical protein secretion, p40 Syt1, a participant of nonclassical FGF1 secretion pathway, comprises only the extravesicular portion of the polypeptide including C2A and C2B [72]. The C2A is a Ca^2+^ binding domain and has been shown to bind Cu^2+^ with nanomolar affinity [73]. Four Cu^2+^ ions have been shown to bind per mole of the C2A domain (Figure 3). Metal competition binding monitored by ITC and the results of Tb^3+^ binding competition experiments showed that three of the four Cu^2+^ ions bound to the C2A can be replaced by titration with Ca^2+^. Paramagnetic NMR spectroscopy studies revealed that the residues in the three apical loops of the C2A domain structure provide binding interface for three of the four Cu^2+^ ions bound [73]. The residues in the C2A domain that contribute to Cu^2+^ binding include Asp172, Gly174, Asp178, Lys182 and Val183 (in loop-1), Lys200 (in loop-2), and Ala227, Asp230, Phe231, Asp232, Phe243, and Asp238 (in loop-3). The fourth Cu^2+^ binding site is provided by Gly253 and His254 located in the unstructured loop linking β-strands VII and VIII [73]. Interestingly, both Gly253 and His254 are bridged by an array of hydrogen bonds to several residues in the structure of the C2A domain.

Cu^2+^ induces the formation of a homodimer of FGF1 through specific oxidation of Cys30. The FGF1 homodimer formation is inhibited in the presence of the anti-inflammatory drug, amlexanox [74]. Based on the results of two-dimensional NMR studies, the Cu^2+^ binding site on FGF1 is located proximally to Leu28, Cys30, His35, His138, Tyr139, Leu147 and Leu149.

The Cu^2+^ binding studies with the protein components of the FGF1 MRC provide a tentative sequence of events leading to the formation of the FGF1 homodimer. The Cu^2+^-binding affinities of the “copper chaperones” and S100A13 are in the similar range suggesting that S100A13 is the direct recipient of Cu^2+^ from the “copper chaperones” [68,73]. The Cu^2+^ bound to S100A13 is possibly then transferred to the C2A domain of p40 Syt1. This notion of the transfer of Cu^2+^ from S100A13 to the C2A domain of p40 Syt1 appears reasonable because: (1) ITC results show that S100A13 and C2A domain of Syt1 interact with each other with moderate affinity [73]; (2) the C2A domain binds to Cu^2+^ with very high affinity in the nanomolar range; and (3) ITC and ^1^H-^15^N HSQC perturbation studies suggest that the C2A domain binds to FGF1 [75]. As FGF1 and the C2A domain of Syt1 are involved in direct interaction, the Cu^2+^ bound to the C2A domain is plausibly utilized to promote the specific oxidation of Cys30 in FGF1 to form the homodimer [73].

## 4. Understanding the Formation of FGF1 MRC

The molecular sequence of events leading to the formation of the FGF1 MRC is still unclear. It is not known whether the protein components of the FGF1 MRC interact with each other in their apo-forms or they bind to Cu^2+^ before they bind to each other. ITC results clearly show that FGF1 and the C2A domain of p40 Syt1 can bind to each other in the absence of Cu^2+^. The interaction is shown to be significantly stronger in the presence of phosphatidylserine (PS) vesicles [75]. Similar binding experiments revealed no binding between apoS100A13 and the C2A domain of Syt1. The three dimensional solution structure of the ternary complex of the FGF1/S100A13/C2A shows domain complex [76]. The residues in S100A13 that bind to FGF1 include, Thr15, Phe21, Thr22, Phe 23, Arg25, Gln, Lys30, and Asn36 and similarly, the S100A13 binding sites on FGF1 are contributed by Lys98, Asn99, Arg112, Arg115, His117, Tyr118, Gly120 and Lys121. Interestingly, FGF1/S100A13 interface does not include the residues at the *C*-terminal end of S100A13 (residues, 88 to 99). This observation is surprising considering the fact that *in vitro* FGF1 release assays, using the *C*-terminal deletion mutant (Δ88-98S100A13) of S100A13, conclusively show that the FGF1 MRC is not organized in the absence of the *C*-terminal fragment of S100A13 [24]. The absence of residues in the *C*-terminal segment (residues, 88–98) of S100A13 in the FGF1/S100A13 binding interface is perplexing because ITC data conclusively suggest that the *C*-terminal deletion mutant (Δ88-98S100A13) has no or little affinity to bind to FGF1. The reported ternary structure of the FGF1/S100A13/C2A domain lacks Cu^2+^ and therefore does not provide sufficient insight on the molecular events leading to the formation of the FGF1 MRC and the role of metal ions in the organization of the FGF1 MRC.

## 5. Phospholipids and Nonclassically Released Proteins

During the cell stress, FGF1, S100A13, p40 Syt1 [77] and Anx 2 [20] translocate to the vicinity of the cell membrane. SphK1 exhibits phosphorylation-dependent association with the cell membrane [78]. The peripheral translocation of FGF1 is stimulated also by thrombin [48]. The staining of nonpermeabilized stressed cells for externalized FGF1 showed that it is not evenly distributed across the cell membrane but concentrated in distinct oval-shaped domains with the diameters from 0.5 to 5 μm [40]. Most of these FGF1-positive domains are also stained for the acidic phospholipid (PL) phosphatidylserine (PS). Similar to other acidic PL, under normal condition PS is asymmetrically distributed to the inner leaflet of the cell membrane [79]. However, cell stress induces the translocation of FGF1 to the outer leaflet of the PL bilayer [80]. The simultaneous externalization of PS and FGF1 was observed also in U937 promonocytic leukemia cells induced to differentiate to macrophages [40]. Interestingly, the knockdown of PL scramblase 1 (PLSCR1), an enzyme involved in PS transmembrane translocation [81], influenced neither the secretion of FGF1 nor PS externalization [40]. However, chemical compounds taurine and isoproterenol, which inhibited PS externalization, suppressed the stress-induced FGF1 externalization [40]. These results suggest a role of acidic PL translocation in the secretion of FGF1. This hypothesis implies the binding of FGF1 and other MRC components to acidic PL. Indeed, FGF1 binds PS (*K*_d_ ≈ 490 nM) through the basic amino acid residues localized in its *C*-terminal portion [53]. The high affinity binding of acidic PL is also characteristic for Syt1 [82], S100A13 [83], SphK1 [84], Anx 2 [85] and FGF2 [86].

Mach and Middaugh proposed to use the liposomes loaded with carboxyfluorescein to study the effects of FGF1 on PL bilayers [87]. In this experimental model, FGF1 destabilized liposomes containing acidic PL and thus induced the release of caroxyfluorescein and burst of fluorescence due to the abrogation of its high concentration-dependent quenching. The same model was later used to demonstrate that IL1α[10], p40 Syt1 and S100A13 [88] also preferentially destabilize the bilayers composed of acidic but not of zwitterionic PL. Mutation analysis has shown that lysine residues 114, 115, 126 and 127 in FGF1 and 326, 327 and 331 in p40 Syt1 are critical for both the destabilization of liposome and nonclassical secretion of these proteins [88].

FGF2 [89] associates with the non-raft domains of the cell membrane. Recently, it has been shown that the formation of FGF2 aggregates on PIP2-containing bilayers induces pore formation [86]. Both nonclassical export of FGF2 from the cells and the FGF2-induced pore formation in artificial membranes require the phosphorylation of tyrosine residue 82 [86,90].

The importance of interactions of signal peptide-less FGFs with acidic PLs for the nonclassical secretion implies that these interactions can be used as targets to regulate the accessibility of FGF1 and FGF2 in the organism. However, to achieve this goal, the molecular mechanisms of these interactions and of their effects on membrane stability need to be understood. A plethora of physical methods has been applied for these studies, and their results are discussed below. In particular, we will consider which structural features of polypeptides are responsible for interactions with PL and what cell-free systems can be used to study the mechanisms of translocation of signal peptide-less proteins through the PL bilayer.

## 6. Determining the Lipid Binding Domains of FGF1 MRC Components

Understanding the lipid interactions with the individual protein components of the FGF1 MRC is critical to trace the mechanism of release of FGF1. There have been several attempts to understand the interaction of FGF1 with PL membranes. Middaugh and coworkers showed that FGF1 in its partially structured state, obtained at low pH, exhibits high binding affinity to membrane vesicles [87,91–94]. Studies from the Gallego group suggested that an acid-induced partially folded state of FGF1 exhibits high binding affinity to PS vesicles [95]. Interestingly, Wiedlocha *et al*. [96], using FGF1 fused to diphtheria toxin, demonstrated that partial unfolding of FGF1 is critical for the translocation of internalized FGF1 from endosomes to cytosol. However, Wesche *et al*. [97] showed that disulfide bonded mutants of FGF1 having near-native like folding also can successfully translocate to cytosol. The experiments using chimeras with dihydrofolate reductase, which can be locked in folded conformation by its inhibitor aminopterin, demonstrated that neither FGF2 [98] nor FGF1 [38] require complete unfolding for their nonclassical release. Rajalingam *et al*., [75] showed that a molten globule-like intermediate is structure realized in FGF1 under acidic conditions in the urea-induced unfolding pathway. Interactions involving Cys30, Gly33, Gly33, Gly34, His35, Phe36, Ser90, Gln91, Asn94, Glu95, Cys97, Glu101, Arg102, Glu104, His107, Thr137, His138, and Ile144 are disrupted in the molten globule-like state. These residues are mostly located in β-strands I, II, VIII, and the loop connecting β-strands VIII and IX, and β-strand X. ITC results revealed that the FGF1 in its molten globule-like state exhibits significantly higher binding to small unilamellar PS vesicles than in the native conformation [75].

The C2A domain of Syt1 also exists in a partially structured state under acidic conditions. 1-anilino napthalene sulfonate binding studies showed that the C2A domain under acidic conditions assumes the molten globule state with clear perturbation of residues (Gln164, Ala165, Ala170, Thr177, Ser178, Lys196, His198, Lys200, Phe212, Lys213, Val214, Glu218, Ser235, and Ile240) located in the unstructured loops connecting the beta-strands in the protein [75]. Interestingly, the binding affinity of the molten globule state of the C2A to PS vesicles is 100-fold greater than in the native conformation. The C2B domain of Syt1 has been shown to exhibit binding affinity to both Cu^2+^ and PS vesicles [99]. The amino acid residues in C2B that contribute to Cu^2+^ binding include β-strand I, Loop I, β-strand IV, Loop II, β-strand V1, and Loop III. Interestingly, some of the residues in Loops I, II and III (Gly306, Ile367, and Lys375), which are a part of the Ca^2+^-binding site in the C2B domain, contribute to binding to PS vesicles.

S100A13, like FGF1, is bereft of the classical signal sequence [100]. It is released spontaneously from transfected NIH 3T3 cells [24]. Coexpression of S100A13 has been shown to facilitate the stress-induced release of FGF1 [24]. Kathir *et al*. [101] demonstrated that apoS100A13 interacts (*K*_d_ ≈ 500 nM) with negatively charged small unilamellar vesicles (SUVs) of PS but not SUVs of phosphatidylglycerol (PG). Interestingly, apoS100A13 undergoes a subtle conformational change upon binding to PS vesicles, which in turn decreases the conformational stability of the protein [101]. In the Ca^2+^-bound state, S100A13 is shown to exhibit weak PS binding affinity and this has been attributed to the decreased solvent-exposed non-polar surface(s) in the holo-protein. NMR studies revealed four residues (Gly31, Leu49, Ser74 and Lys85) to be located at the lipid binding interface [101]. Results of this study indicate that the binding affinity of apoS100A13 plausibly aids in the anchoring of the FGF1 MRC to the cell membrane.

Based on the reported Cu^2+^ and lipid binding affinities, it appears that three of the known protein components of the FGF1 release complex (FGF1, S100A13 and p40 Syt1) play important roles in the translocation of FGF1 across the cell membrane. Although clear experimental data on the chronology of molecular events leading to the release of FGF1 in to the extracellular compartment are missing, available experimental results plausibly suggest that interaction of FGF1 with the p40 Syt1 C2A domain is the first significant molecular event that occurs in the non-classical release pathway of FGF1. The more acidic microenvironment prevailing in the proximity of the plasma membrane can be expected to populate molten globule-like states of FGF1 and the C2A domain of Syt1. This proposal seems reasonable because Rajalingam *et al*. [75] showed that FGF1/C2A domain interaction is much tighter in their molten globule-like states. The interaction of Cu^2+^-bound S100A13 with the FGF1/C2A domain appears to be the next logical molecular event and S100A13 appears to provide a binding interface for the anchoring of the multiprotein release complex with membrane bound Anx 2. S100 family of proteins is known to interact with those belonging to the annexin family [102]. Anx 2, in particular, exists on the inner and outer sides of the plasma membrane [103]. It is reported to “flip-flop” between the inner and outer sides of the cell membrane after thrombin treatment and under stress conditions [104]. Therefore, it is possible that the “flip-flopping” property of Anx 2 facilitates the export of FGF1 into the extracellular compartment. However, it is still not clear if FGF1 alone or the whole FGF1 MRC is exported into the extracellular medium. Intensive research is ongoing to elucidate the interplay of molecular forces, which operate in the non-classical release of FGF1. Particularly, the efforts are focused on the understanding of the translocation of FGF1 MRC components across the plasma membrane.

## 7. Methods to Study the Interactions of Nonclassically Released Proteins with Membranes

Molecular mechanisms of signal peptide-less protein translocation across the cell membrane remain enigmatic. The studies of these mechanisms require the use and further development of methods allowing detection of the protein-induced perturbation of cell membranes and protein penetration across the PL bilayers. They also necessitate the design of cell-free models, in which the application of sensitive detection methods would not be hampered by “cell noise”.

### 7.1. Sum Frequency Generation Vibrational Spectroscopy

**Figure 4:**
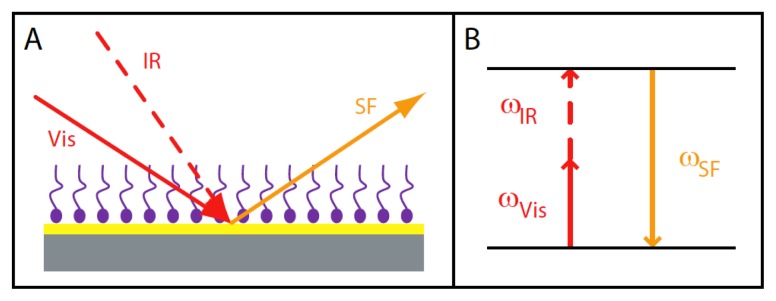
Sum frequency generation scheme. (**A**) Sum frequency generation occurs at an interface when two pulsed laser beams interact; (**B**) The SF frequency emitted is at the sum of the visible and the infrared incident frequencies (ω_Vis_ + ω_IR_ = ω_SF_).

Information regarding the changes in conformational order and orientation of the PL comprising the membrane is critical for the study of the nonclassical protein export. A spectroscopic technique uniquely capable of elucidating both conformational order and molecular orientation is sum frequency generation vibrational spectroscopy (SFS). Unlike typical spectroscopic methods, SFS records vibrational spectra of exclusively interfacial molecules [105,106]. SFS utilizes the nonlinear optical phenomenon of sum frequency generation (SFG). SFG occurs when light interacts at an interface under specific symmetry and phase matching conditions [105]. SFG is typically generated by two pulsed, high-energy lasers that are temporally and spatially overlapped at an interface. The pump beam has a fixed visible frequency, while the probe beam has a tunable infrared frequency (Figure 4a). As the two incident laser beams interact at an interface, light is generated and emitted from the interface at the sum of the two incident frequencies such that ω_Vis_ + ω_IR_ = ω_SF_ (Figure 4b).

Molecules in bulk (that is non-interfacial molecules) do not produce an SF signal since they are isotropically distributed and possess inversion symmetry. Conversely ordered molecules at an interface typically lack inversion symmetry and therefore produce an SF signal. Consequently, interfacial molecules may be probed via SFS without spectral contributions from the bulk [107]. The emitted SF signal is resonantly enhanced when the frequency of the infrared beam matches a vibrational mode of the interfacial molecules. Thus, the vibrational spectrum of the interfacial molecules is measured as a function of the infrared frequency; however, SFG inherently up-shifts the emitted signal to visible wavelengths [105]. SF spectra provide two forms of molecular information: the degree of conformational order, and the orientation of the molecules at the interface (however, the latter requires a non-resonant background signal from the substrate). The conformational order of the interfacial molecules may be determined by the relative strength of various vibrational modes. For example, if a highly ordered, fully trans monolayer of PL at an interface were probed, solely methyl group resonances would be present in the spectrum. This arises from the fact that the methyl groups that terminate the alkyl chain tails of the PL break inversion symmetry and hence are SF active. Conversely, the methylene groups comprising the PL tails possess inversion symmetry and therefore are SF inactive. Should alkyl chain disorder be introduced, producing gauche defects in the PL tails, the methylene group resonances would lose inversion symmetry and become SF active. As such, comparison of the methyl and methylene resonance intensities for the fully ordered monolayer and the disordered monolayer provides the degree of conformational order of the interfacial molecules [108]. Further, the orientation of the interfacial molecules may be determined from the phase of the SF spectrum (“peaks” *vs*. “dips” relative to the baseline), provided that the substrate supporting the molecules produces a non-resonant background as a reference.

The first SF spectra were recorded in 1987 by Shen *et al*. and shortly after by Harris *et al*. [109–111]. Since this pioneering work, SFS has been applied to many synthetic and biological systems [105]. Current biological applications vary widely, but include applications of SFS to model membrane systems, and protein transport studies. The Richmond group, at the University of Oregon, has extensively studied surfactant adsorption at the water/oil interface and PL at both the water/oil and air/water interfaces, probing both the conformation of the various lipids and their effect on solvent structure [108,112–118]. Further, Wolfrum and co-workers have combined SFS studies of PL with Fourier transform infrared spectroscopy to probe the fluidity of drop cast lipids on solid substrates, specifically assessing the order of the hydrocarbon chains [119,120]. The Bonn group has performed numerous SFS studies on various PL monolayers at the air/water interface, specifically exploring the effect of surface pressure, [121,122] the presence of calcium and sodium ions in the subphase [123], and separately, DNA in the subphase [124] on PL ordering. Bonn *et al*. have also probed the interaction of the PL monolayer with the peptide duramycin [125]. These and other studies have also yielded information regarding the structure of water in proximity to the PL [126–130]. In addition, Bonn has investigated the effect of including cholesterol in the monolayer, probing the ordering of the monolayer via SFS [131]. Cholesterol-induced PL condensation has also been explored by Itoh *et al*. [130]. Furthermore, Itoh and co-workers performed SFS studies on mixed PL monolayers, determining the phase transitions of the monolayers with increased surface pressure, and separately, the interaction of the monolayer with polymyxin B, an antibiotic [132,133]. The Yan group has probed PG monolayers at the air/water interface [133] and studied the interaction of human islet amyloid polypeptide with the lipid monolayer [134]. Recently, Viswanath *et al*. studied the interaction of thiocyanate ions with zwitterionic PL monolayers [135], and Hill *et al*. assessed an isoniazide peptide conjugate’s affinity for zwitterionic PL monolayers [136]; both studies were performed at the air/water interface.

The Rutland group has probed the conformational order of PL based on the degree of unsaturation as well as on variations in the headgroup (including selective deuteration) at the air/aqueous solution interface [137,138]. Measurement and simulation of lung PL and surfactant distributions have been studied by Ma and Allen, specifically investigating the conformation of PC monolayers as a function of lateral pressure at the air/water interface [139–141]. Ma and Allen demonstrated for the first time that SFS could be employed to study the hydration state of PL head groups [142,143], and further have studied the hydration and orientation of the phosphate group in PC monolayers [144], and the interaction of PC monolayers with dimethylsulfoxide [145]. Additionally, the Allen group, as well as the Walker group, have examined the interaction of anionic [146,147] and cationic [147] surfactants with PC monolayers at the air/water interface. Similarly, the conformation of mixed lipid and surfactant monolayers at the air/water interface has been probed by the Miller group, and the conformational information correlated with interfacial water structure [148]. Further studies have also employed various PL at the air/water interface to study interfacial water [149,150].

In addition to the air/water monolayer studies described above, many SF studies have been performed on PL bilayer systems. The Conboy group has studied the conformation of PL comprising each leaflet of bilayers supported on solid structures [151]. In addition, Conboy and coworkers have assessed the phase transition temperature [152], phase segregation behavior [153,154], the binding affinity of various drugs to PL membranes [155], and the transbilayer movement, or flip-flop, of PL in bilayer systems [156–160]. Conboy *et al*. have also studied PL systems in the presence of various membrane-spanning peptides such as gramicidin A [160], WALP and melittin [161], and separately, external electrostatic forces [162]. Tong *et al*. have also employed solid supported membranes to study the effect of phospholipase A1 on membrane hydrolysis [163]. Kim *et al*. studied the structure of interfacial water in relation to solid supported membranes [164]. Hybrid bilayer membranes (HBMs) have also been investigated by SFS. Petralli-Mallow and Briggman *et al*. have studied vesicle fusion and Langmuir-Blodgett deposition onto self-assembled alkanethiol monolayers, probing the phase transition temperature of the fabricated membranes, and investigating the interaction between cholesterol and PL in the HBM [165–168]. Davies and co-workers have explored the effect of deposition surface pressure on the ordering of PL and alkanethiol monolayers, with and without cholesterol present [168,169]. Lis *et al*. have probed vibrations in the head group of PL deposited by the Langmuir Schaefer technique on self-assembled monolayers (SAMs) [170]. SFS has also been employed to study the conformational changes that occur in membranes in the presence of proteins or polysaccharides. A study by the Neivandt group was the first to demonstrate protein-induced deformation of a PL membrane (a HBM) using SFS [171]. The Chen group has examined membrane perturbations of solid supported bilayers induced by antimicrobial peptides [172–175], membrane-active polymers [174], melittin [176], and magainin [177]. Recently, Chen and co-workers have expanded to cushioned supported bilayers, specifically investigating poly(l-lactic acid) (PLLA) supported PG bilayers and their interaction with Cecropin P1, an antimicrobial peptide [178].Chen and co-workers have further probed the orientation of the G protein β subunit [179], melittin [180], antimicrobial peptides [175,179–182], alamethicin [183], tachyplesin I [184], and cytochrome b5 [185], embedded within various bilayers. Additionally, the Miranda group studied the interaction of polysaccharides with phosphatidic acid [186,187], and separately, with PL [188].

The work described above provides an overview of the utilization of SFS to study PL membranes, an area of interest that continues to expand in the growing SF community. While fundamental questions are being addressed, it is noteworthy that the vast majority of studies do not involve live cells. The high power, pulsed lasers required for SFS are prone to irreversibly damage cells. Additionally, interpretation of a live cell SFS spectrum is complicated due to the various components found in the cellular membrane that can contribute to a measured spectrum. For example, Inoue *et al*. have constructed a non-scanning vibrational sum frequency generation microscope capable of collecting SF signal from an onion root cell [189]. While they were successful in collecting spectra, they were not able to clearly assign spectral resonances to cellular constituents [189]. As such, it is most often necessary to employ model membrane systems in SFS studies, and it is critical that the systems be physiologically relevant. However, while the model membrane systems utilized previously have given new insight into membrane function, they are not amenable to protein transport studies. Membranes on solid substrates clearly do not allow for protein transport, while membranes at the air/water or oil/water interface are not at the aqueous/aqueous interface necessary for physiological relevance. Consequently, there exists a pressing need for an alternative model membrane system that allows for protein transport and is physiologically relevant.

### 7.2. Fluorescence Correlation Spectroscopy

**Figure 5:**
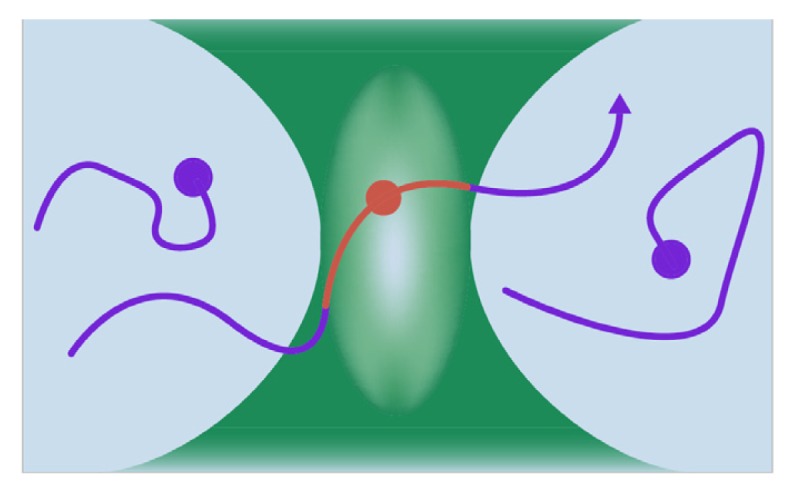
Fluorescence correlation spectroscopy scheme showing diffusion of fluorophores through the observation volume.

Employing model membrane systems as platforms for studying protein transport requires characterization of the diffusion coefficient of component PL to ensure physiologic relevance. Such information may be obtained via fluorescence correlation spectroscopy (FCS). Developed in the 1970s [190], FCS is a single molecule technique that yields molecular diffusion coefficients, chemical rate conversions, and photophysical information [191–193]. FCS inherently requires the incorporation of a low concentration of probe molecules in the membrane. As the probe molecules, typically fluorophores, diffuse through an observation volume created by a tightly focused laser beam, they fluoresce (Figure 5). The fluorescence emission is collected as a function of time to produce a time course, and the time course is mathematically related to itself resulting in a correlation curve [194]. The correlation curve may subsequently be fit to a given diffusion model [191].

The instrumentation required for such measurements may be as simple as an excitation laser source, a confocal microscope with appropriate filters and collection optics, and an avalanche photodiode (APD) or photomultiplier tube (PMT) for detection. Measurements are facilitated by the use of automated hardware that converts the intensity time course into a correlation curve (a correlator card) [195]. The use of FCS for membrane characterization has been extensive, with studies on live cells, vesicles, and planar membranes [196–198]. When fabricating a PL bilayer for examination by FCS, a fluorescent PL analog, such as 1,1′-dioctadecyl-3,3,3′,3′-tetramethylindocarbocyanine perchlorate (DiI C18) or a fluorescently tagged PL, such as *N*-(7-nitrobenz-2-oxa-1,3-diazol-4-yl)-1,2-dihexadecanoyl-*sn*-glycero-3-phosphoethanolamine, triethylammonium salt (NBD-PE), is typically added to the PL mixture. Incorporation of the probe molecule may also be achieved by incubating cells or membranes with the fluorophore [196,197]. The concentration of fluorophore required for FCS membrane measurements is of the order of 0.001–0.01 mol% of fluorophore to PL, which is considerably lower than for other fluorescence techniques, e.g., fluorescence recovery after photobleaching (FRAP) [198]. Reducing the amount of fluorophore in the model membrane decreases the potential for perturbation of the membrane, thereby increasing the physiological relevance of the measurement. Ideally, the fluorophore chosen for membrane studies should have a high quantum efficiency, a high fluorescence quantum yield, low triplet activity (an intermediate transition during relaxation occurring on fast time scales), and should not photobleach readily, in addition to being a lipid analog [193,199].

As noted above, application of FCS to membrane studies has been extensive. Of note, Elliot *et al*. spread PL monolayers on polystyrene films, and studied PL lateral diffusion via FCS [200]. The Webb and Schwille groups, independently and collaboratively, have performed a variety of FCS studies on live cells and vesicles. Comparisons were made of fluorophore diffusion in live cell membranes and model membrane vesicles. Separately, fluorophore diffusion in model membranes has been employed to probe the effect of phase separation and cholesterol addition [196,201–204]. Additionally, both groups utilized supported PL bilayers to study membrane PL distribution and dynamics via FCS [194,205,206]. Zhang and Granick have employed FCS to investigate diffusion in each leaflet of solid supported membranes, and separately, monitored the effect of an adsorbed polymer on lipid diffusion [198,207]. Further, FCS has been used to probe membrane-protein interactions, with an emphasis on lipid domains (rafts) in supported bilayers [194,197]. Proteins are involved in raft formation, and therefore tracking protein movement optically, in addition to monitoring the movement of the lipid domains via a fluorescent lipid analogs, provides a powerful methodology to study raft formation [202]. Vesicle model membranes and fluorescently tagged proteins have been utilized to study protein diffusion in model membranes with FCS. For example, the Schwille group studied the raft-associated protein, human placental alkaline phosphatase, monitoring its change in diffusion rate as it associated with domains in the model membrane [202]. The Kinjo group employed PS liposomes to study the binding interaction of lipids with a rhodamine labeled protein known as protein 4.1 [202]. FCS measurements of rhodamine 6G, protein 4.1, and protein 4.1-liposome complexes formed either by contact with PS or with PC liposomes, were performed. The resulting correlation curves had significantly different shapes with variation in molecular weight and interaction between the protein and lipids [202]. Indeed, it was demonstrated that the protein of interest only interacted with PS liposomes and not with PC liposomes [202]. A study by Campbell *et al*. of polychlorinated biphenyl (PCB) interactions with model membranes further demonstrated the power and capabilities of FCS [208]. Specifically, supported bilayers incorporating fluorescently tagged lipid molecules were fabricated, and the diffusion characteristics of the lipids determined via FCS. Subsequently, two different fluorescently active PCBs were brought into contact with tagged and non-tagged bilayers, and each PCB’s correlation curve measured. Fitting of each PCB’s correlation curve resulted in two diffusion coefficients, one value within error of the PL diffusion coefficient, and one value an order of magnitude slower, indicating two potential interactions of each PCB and the membrane [208].

The studies presented above are a small sampling of the many membrane and membrane-protein FCS studies to date. It is important to note that all of these studies were calibration-dependent, that is, required the use of a fluorophore with a previously determined diffusion coefficient. The diffusion coefficient was either determined via a separate measurement technique, or had been previously reported in the literature. Unfortunately, many groups have found FCS to be sensitive to experimental fluctuations, and further, reported values of diffusion coefficients of commonly employed fluorophores in the literature often differ significantly [209–211]. To this end a derivative of confocal FCS has recently been developed that requires no calibration standard [212]. Termed “z-scan” FCS, the technique utilizes the parabolic dependence of the characteristic diffusion time and effective particle number (as determined in standard FCS measurements) on the z-axis position of the sample in the observation volume to determine the diffusion coefficient and concentration. The technique is uniquely suited to planar systems, such as model membrane systems, and has been employed to study solid supported membranes formed by vesicle fusion [212–218], giant unilamellar vesicles (GUVs), [194,210,219–221] and the plasma membrane and membrane-protein interactions of live cells [222,223]. Since z-scan FCS is ideally suited to, and limited to, planar systems, it is an excellent complementary technique to SFS for the study of non-classical protein secretion employing model membrane systems.

## 8. Model Membrane Systems to Study the Nonclassical Protein Release

Model membranes are artificial lipid bilayers that are either spherical or planar, and may be freely suspended or supported on a solid substrate (either directly or via a cushion). Select membranes potentially allow proteins to be incorporated into, or translocated through, the bilayer. A model membrane provides a simplified version of a plasma membrane and is often more amenable to microscopy or spectroscopy. The various model membrane systems are reviewed below, with emphasis on those most amenable to the study of non-classical protein release, and application of SFS and/or FCS.

### 8.1. Vesicles and Liposomes

Most cytoplasmic organelles may be considered membrane vesicles of various sizes. For example, Golgi-derived exocytotic vesicles are key elements of the classical secretion pathway and have an approximate radius of 100 nm [224]. Artificial membrane vesicles are spherical structures comprising a PL bilayer surrounding an aqueous core and are stable in aqueous solution. Artificial vesicles are also termed liposomes; indeed, the terminology for vesicles and liposomes is often used interchangeably. However, it should be noted that vesicles may also be formed from surfactants. PL vesicles or liposomes are often characterized according to their diameters, for example small unilamellar vesicles (SUVs), large unilamellar vesicles (LUVs), and giant unilamellar vesicles (GUVs) with radii of 4–20 nm, 50 nm–10 μm, and >10 μm, respectively are named for their sizes [225]. Additionally, liposomes may be described by the number of PL bilayers they are composed of (uni- or multilamellar vesicles) and the charge of the outer surface (anionic, cationic, or neutral) [225]. Due to the enclosed bilayer structure of vesicles, they represent an excellent model of the plasma membrane of a cell, providing a model for studying membrane properties as well as membrane-protein interactions [10,88,226]. Additionally, vesicles and liposomes often serve as intermediates for producing other model membrane systems [108,165,227]. Due to the breadth of research utilizing this particular model membrane system, a selection of studies directly related to non-classical protein transport are reviewed below.

An assay utilizing “inside-out” vesicles formed from reconstituted plasma membranes of cells was developed by Nickel *et al*. [37]. The assay established a method for membrane extraction as well as characterization by affinity purification of the “inside-out” and “right side-out” vesicles formed [37]. Transmembrane transport of FGF2 was studied by incubating the vesicles with the protein at 37 °C for 4 h. After removing excess protein via rinsing, a protection study was performed using a protease and or detergent. Results were visualized by Western blot after running SDS-PAGE. It was demonstrated that FGF2 was capable of entering the vesicles as evidenced by protection from the protease [37]. A similar method has been used to assess the transmembrane transport behavior of somatostatin [228].

Artificial liposomes have been employed by Prudovsky and co-workers to study the ability of FGF1 MRC members to destabilize PL membranes [88]. Unilamellar liposomes were prepared from a variety of synthetic PL (PS, PI, PG, and PC) in an aqueous solution containing carboxyfluorescein, a fluorophore. After removing fluorophore external to the liposomes employing a dextran desalting column, the fluorophore containing liposomes were monitored via spectrofluorimeter [88]. At a temperature representative of heat shock, FGF1, p40 Syt1, and S100A13 were added separately to the liposome solutions, and changes in fluorescence intensity monitored. An increase in fluorescence intensity indicated release of carboxyfluorescein from the liposomes and the concomitant removal of the fluorophore from a self-quenched state. It was demonstrated that FGF1 most efficiently destabilized membranes comprising acidic PL (PI, PG, PS), but was ineffective at destabilizing zwitterionic PC liposomes. S100A13 behaved in a similar manner to FGF1. However, p40 Syt1 only destabilized liposomes comprising PI, thereby indicating a degree of selectivity [88]. These findings reinforce the importance of determining the role of the PL bilayer in secretion of signal peptide-less proteins.

While vesicles and liposomes are physiologically relevant due to their shape and ability to incorporate or translocate proteins, they are not suited for use in SFS studies. As described above, SFS is typically restricted to planar interfaces; a sphere of lipids would likely provide multiple interfaces and additionally would not maintain a stationary position during spectral acquisition. Although SFS studies have been performed in a scattering geometry from spheres in a solvent, the signal was extremely weak and the experiment was limited to a carefully selected sphere/solvent pair [229,230]. Since commonly employed SFS geometries, such as external reflection and total internal reflection, have been readily applied to planar lipid membranes as reviewed above, the following presents an overview of planar model membrane systems.

### 8.2. Black Lipid Membranes

An early generation model membrane system, in use since the 1960s, is the black lipid membrane (BLM) [231,232]. A BLM is a PL bilayer formed across an aperture maintained in aqueous solution. This type of membrane was first formed for use in patch clamp experiments, however, the ease of forming planar bilayers across small holes has greatly improved with the development of microfabrication techniques [233]. Various studies have been performed on BLMs including ion channel investigations by monitoring electric potential, investigation of chemicals binding to receptors maintained in the membrane, and studying the interaction of DNA with membranes [231]. However, the stability of the BLM is problematic. Indeed, the membranes typically only last for a few hours [233]. To combat this issue, agarose, a hydrated polymer, has been used to support and stabilize BLMs with minimal detrimental effects observed on the membrane properties [233].

### 8.3. Hybrid Bilayer Membranes

**Figure 6:**
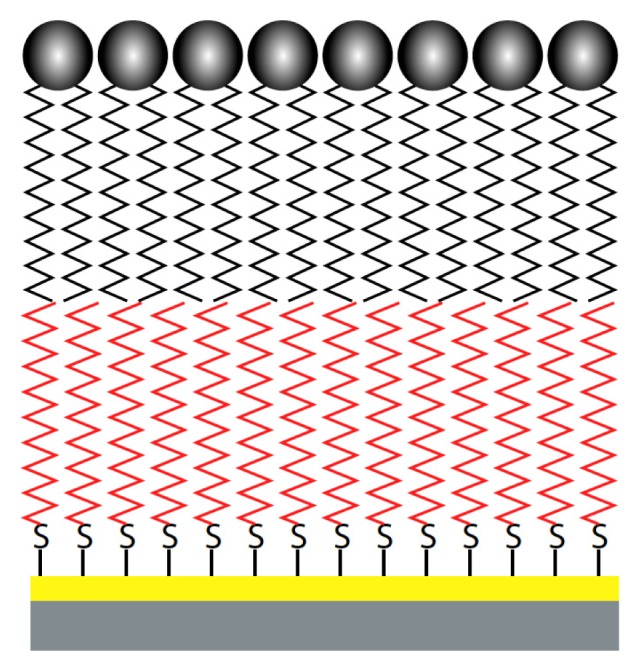
Hybrid bilayer membrane. The lower layer (red) represents the alkanethiol, while the upper layer (black) represents the pL.

A more stable alternative to BLMs are hybrid bilayer membranes (HBMs). HBMs consist of a solid support functionalized with a hydrophobic monolayer, with a second monolayer composed of PL deposited on the hydrophobic layer (Figure 6) [234,235].

The substrate is most often a metal surface, e.g., a gold coated silicon wafer, on which an alkanethiol monolayer may self assemble and covalently bind. The alkanethiol is dissolved in a solvent, e.g., methanol or ethanol, and the substrate placed in solution, allowing self-assembly to take place. The quality of the monolayer may be assessed by contact angle measurements [236]. The subsequent formation of the PL monolayer may be via vesicle fusion, Langmuir-Blodgett (LB) deposition, or Langmuir Schaefer (LS) deposition. Vesicle fusion occurs via spontaneous rupture and spreading of PL vesicles on the hydrophobic alkanethiol monolayer. Alternatively, LB and LS deposition employ a trough containing an aqueous subphase on which a monolayer of PL is spread, drop-wise, from a volatile, non-aqueous solvent. The PL spontaneously orient such that their hydrophilic head groups are immersed in the water subphase, while their hydrophobic tails are positioned into the air [234,237,238].After solvent evaporation, the PL are compacted via a closing barrier system on the trough to a specified surface pressure, (measured by a Wilhelmy film balance). Finally, the substrate of the HBM with its alkanethiol monolayer is passed vertically for LB deposition, or horizontally for LS deposition, into the subphase, through the PL monolayer, resulting in the deposition of an alkanethiol supported lipid leaflet. HBMs are an attractive experimental system in a variety of ways. Due to their metallic surface, electrochemical techniques and surface plasmon resonance may be employed to characterize the membrane [235]. Additionally, the use of various spectroscopic and microscopic techniques such as atomic force microscopy, ellipsometry, SFS and FCS are greatly facilitated by the planar and stable nature of the membrane [235]. Two studies in particular highlight the utility of HBMs in membrane and membrane protein interaction investigations.

The Petralli-Mallow group and later, the Briggman group have been leaders in the use of HBMs, particularly in SFS studies [165–167,239]. In one particular study, the formation of an HBM via vesicle fusion onto an alkanethiol functionalized gold surface was monitored by SFS [165]. Due to the inversion symmetry required for an interface to be SF active, an HBM had to be constructed that would facilitate spectral differentiation between the alkanethiol layer and the fused vesicle layer. This was achieved through use of a perdeuterated octadecanethiol (d-ODT), and a PC with a perdeuterated headgroup but perprotonated tails. The broadband IR pulses were tuned to the C–H stretching region (2700–3100 cm^−1^). Thus, the resonances detected were from the perprotonated terminal methyl and the methylene groups composing the PL tails. SF spectra revealed that as vesicles were flown through the liquid cell, into contact with the d-ODT monolayer on the substrate, there was an increase in the intensity of the methyl resonances from the PL with time. The contribution of methylene resonances to the SF spectra was minimal. Further, the intensity of the methyl resonances increased after rinsing away the excess and partially fused vesicles, indicating that the PL layer was very well ordered. This work provided a new method for researchers to study HBMs *in situ* with SFS [165].

**Figure 7:**
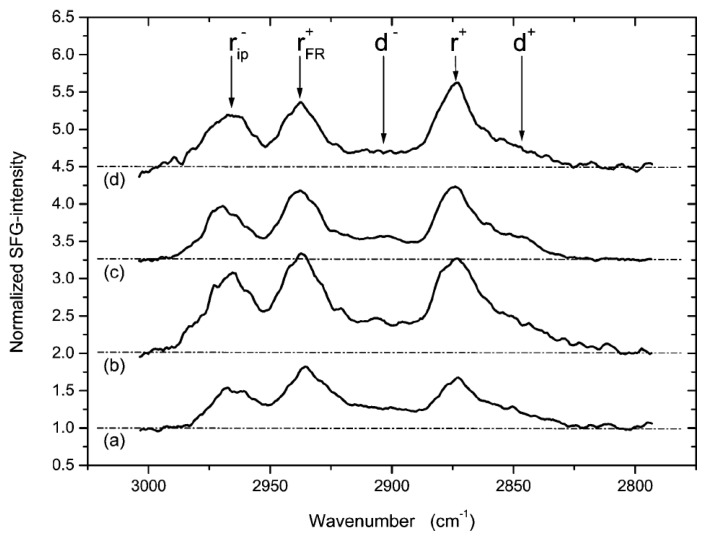
HBM-FGF1 interaction as determined by SFS [171]. *In situ* SF spectra of (**a**) PG vesicle fusion; (**b**) rinsing of excess lipids; (**c**) equilibration with FGF1; (**d**) removal of FGF1, where r^+^ is the symmetric methyl stretch, r_FR_^+^ is the Fermi resonance of the symmetric methyl stretch, r_ip_^−^ is the in-plane methyl asymmetric stretch, d^+^ is the symmetric methylene stretch, and d^−^ is the asymmetric methylene stretch.

Indeed, building upon this study, an investigation of the interaction between FGF1 and an HBM, employing SFS, was performed by Doyle *et al*. [171]. Specifically, the degree of lipid conformational order was measured *in situ* at 60 °C in the presence of FGF1. The HBM constructed consisted of d-ODT self-assembled on titanium primed, gold coated silicon wafers. After mounting the sample in a liquid cell, SF spectra were collected in the C-H stretching region as phosphatidylglycerol (PG) vesicles were flown through the system (Figure 7a). As in the previous study, the only source of SF signal was from the PL. An SF spectrum collected after vesicle fusion to the surface clearly showed strong methyl resonances at 2881 cm^−1^, 2943 cm^−1^, and 2973 cm^−1^, implying that the fused vesicles had created a very highly ordered PL monolayer. Spectral fitting revealed the presence of very weak methylene resonances present at 2860 cm^−1^ and from 2890 to 2930 cm^−1^. The weakness of the methylene resonances in comparison to the methyl resonances indicated that the PG possessed almost complete inversion symmetry, meaning that the PL tails were in a nearly fully *trans* conformation. Additionally, the use of gold as the substrate facilitated the determination of the orientation of the PL from the spectral phase; the PL were found to be oriented with their alkyl chain tails toward the d-ODT surface. Subsequently, any partially fused vesicles and remaining whole vesicles were rinsed away, and a second SF spectrum recorded (Figure 7b). A similar increase in the intensity of the methyl resonances and decrease in the intensity of the methylene resonances was observed to that reported by Petralli-Mallow *et al*. [165], implying a further increase in the order of the HBM [171]. FGF1 was then introduced to the liquid cell containing the PG membrane and permitted to equilibrate prior to another SF spectrum being recorded (Figure 7c). Comparison of the new spectrum with those previously recorded revealed a decrease in the intensity of the methyl resonances and a concurrent increase in the methylene resonance intensities. This result was attributed to FGF1 interacting with the membrane and causing the PL methyl groups to lose symmetry and become more disordered and therefore less SF active. In addition, the methylene groups, which previously were in a nearly fully trans conformation, had more gauche defects present in the PL tails as a result of FGF1 interaction, thereby breaking inversion symmetry and resulting in increased SF activity. It was concluded that FGF1 induced conformational disorder in the PL membrane [171]. The last component of the experiment was to rinse away the FGF1, and record an SF spectrum (Figure 7d). The final spectrum was comparable to that of the HBM prior to FGF1 addition, implying that PG returned to a highly ordered, near fully *trans* conformation, indicating reversibility in membrane deformation [171]. These findings are consistent with the liposome study discussed above where it was demonstrated that FGF1 destabilizes PL membranes [48]. This SFS study was the first to show reversible deformation of membranes by FGF1. As a control, α-chymotrypsin, a protein secreted through the classical pathway and unable to permeabilize membranes composed of acidic PL [87] was employed. Unlike FGF1, it did not induce any significant changes in the membrane as reported by the SF spectra.

It may be concluded from the work of Doyle *et al*. that HBMs provide an amenable membrane model for studies utilizing SFS. However, HBMs possess several limitations. First, the use of an alkanethiol monolayer does not make the HBM a true PL bilayer. Second, since the alkanethiol molecules are covalently bound to the surface, protein transport across the membrane is not possible. As such, the result of membrane-protein interactions can only be assessed from perturbation of the outer leaflet. The ability to create a model membrane in which both leaflets contain PL addresses the first of these limitations and is discussed below.

### 8.4. Solid Supported Membranes

A PL bilayer supported on a hydrophilic substrate is a very satisfactory and widely used model membrane system. Such membranes have been employed extensively for studies not limited to but including channel formation, energy conversion, molecular recognition, and antibody-antigen binding [240–246]. As with the HBM, the planar bilayer facilitates the use of spectroscopic and microscopic techniques (e.g., SFS and FCS), and a greater degree of stability is achieved over BLMs due to the proximity of the solid support. The membrane is separated from the substrate by a 10–20 Å water layer, permitting lateral movement of lipids in the proximal (lower) leaflet [247]. While a solid supported membrane is a step closer to mimicking the plasma membrane, the thin water layer prevents incorporation of transmembrane proteins containing intracellular components, and transport of proteins across the bilayer. In only a few studies have researchers retained protein activity after incorporation into a planar bilayer system, where typically the protein is immobile due to interaction with the solid surface [247–249]. Despite the limitations regarding protein incorporation and transport, SFS has been widely used to study this type of model membrane, in particular the flip-flop of PL between leaflets, as well as membrane-protein interactions (reviewed above).

**Figure 8:**
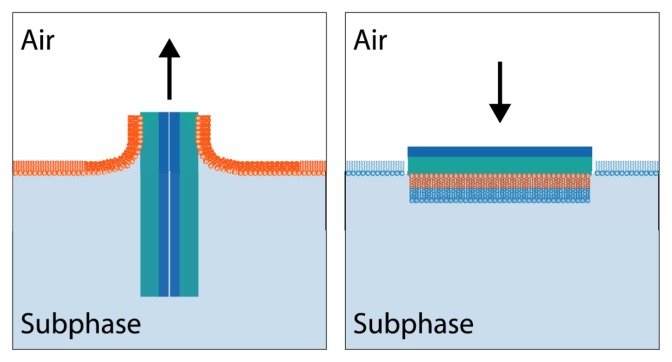
The Langmuir-Blodgett/Langmuir Schaefer deposition technique for lipid bilayer creation. Note that the asymmetric bilayer (represented by orange and separately blue lipids) is readily formed.

Planar supported PL bilayers are primarily formed by two methods: vesicle fusion, and the Langmuir-Blodgett/Langmuir Schaefer (LB/LS) technique [250]. Vesicle fusion, as described previously, employs vesicle rupture to create PL films on solid surfaces. In order to create a bilayer on a hydrophilic surface (rather than a monolayer on a hydrophobic surface for an HBM), liposomes are simply brought into contact with the substrate and allowed to fuse. While simple, vesicle fusion does not allow for control of the PL content of each leaflet of the bilayer, or lipid density. Conversely, the LB/LS technique provides great control over the contents and conformation of each lipid bilayer leaflet [151]. Specifically, the first layer is deposited by the LB technique, described above, but with the substrate initially immersed in the subphase. The second leaflet is deposited by holding the sample above the subphase horizontally, and passing it through into the subphase (Figure 8). The two separate PL depositions allow the monolayer composition of the two leaflets to be varied by replacing the PL monolayer at the air/water interface between the two depositions. Thus, asymmetric bilayers (amenable to SFS studies) may be readily created, for example, membranes may contain perdeuterated PL in one leaflet and perprotonated PL in the other leaflet [153,172].

### 8.5. Cushioned Membranes

**Figure 9:**
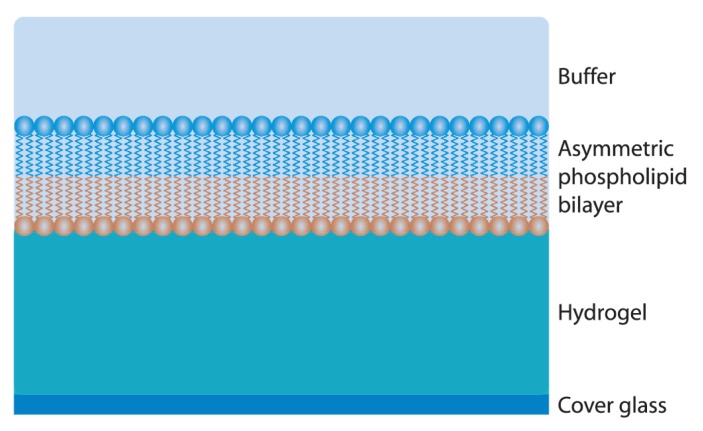
Cushioned model membrane with a hydrated polymer/hydrogel cushion supporting an asymmetric PL membrane.

In order to combat the limitations regarding protein incorporation and protein transport associated with solid supported bilayer membranes, cushioned model membranes were developed. Cushioned model membranes are constructed such that the PL bilayer is separated from the solid substrate by a lipopolymer tether, or a hydrated polymer/ hydrogel layer (Figure 9). This provides space beneath the membrane for incorporation of transmembrane proteins, or to permit protein transport. Many researchers have developed different versions of these cushioned bilayers, notably, Ringsdorf [251], Sackmann [252,253], Israelachivili [227], Offenhausser [254], Tanaka [253,255], Cremer [256–259], Wirth [260], Knoll [261–266], and Frank [265,267,268], among others.

As these model membranes have been created to facilitate many kinds of studies, the size of the space created by the tether, or the more commonly used hydrated polymer, must be considered for protein incorporation or transport. Knowing the size of the intracellular domains of the transmembrane protein of interest is essential, and it is beneficial to have flexibility in cushion thickness. The hydrated polymers and hydrogels used thus far have included polyethyleneimine [227,251,269], polyacrylamide [260,270,271], and polysaccharides such as dextran, cellulose, and chitosan [254,272–274]. A variety of techniques have been utilized to apply the hydrated polymer/hydrogel to the solid support. Adsorption of a variety of polyelectrolytes has been effective on different substrates such as quartz, mica, and silica; however this method has an inherent lack of control over the thickness of the polymer layer. [227,240,251]. Another method employed to form the hydrated polymer layer is LB deposition. Films formed via this technique have well-controlled thickness as the substrate may make multiple passes through a hydrophobic polymer at the air/water interface. Once deposited on the substrate, the polymer must be chemically modified to produce a hydrophilic hydrated layer on which the lipid membrane may be formed [251]. In this particular method, the hydrophobically modified polymer must be synthesized, therefore limiting the application of the technique. A final method that has been used in many applications is spin coating. Briefly, the solid substrate is held, most often by vacuum, on a spinning chuck. The polymer solution of interest is dispensed onto the surface either prior to spinning or during spinning, and after spinning has commenced the polymer solution is spread across and off the surface, leaving a thin film. It is noted that the spin coating parameters can be adjusted to change the thickness of the polymer film. Poly(ethoxaline) and poly(ethoxaline-co-ethylenimine) have both been spin cast onto benzophenone-silane modified silicon surfaces, followed by development under ultraviolet light to covalently bind the polymer to the surface [265]. Perhaps most noteworthy, cushioned systems have been fabricated with the polysaccharide chitosan [254]. Chitosan is easily prepared in an aqueous solution and has been demonstrated to make very controllable and high quality thin films via spin coating [275].

Once the hydrated polymer film has been formed, either LB/LS deposition or vesicle fusion methods may be used to fabricate the PL bilayer. The relative advantages of the two techniques for bilayer formation on hydrated polymer films have been explored using polyethylenimine coated substrates and PC lipids [227]. While vesicle fusion was established as the simplest method to deposit a bilayer on a cushion [227], LB/LS deposition, due to increased control over the bilayer constituents, has since proven to be the most successful bilayer deposition method for a variety of cushions [254,276,277]. Wong *et al*. showed that successful bilayer membrane deposition depended heavily on the supporting substrate’s degree of swelling [227]. Smith *et al*. determined that the roughness of the polymer layer is critical in maintaining membrane fluidity and the mobility of transmembrane proteins [260]. Additionally, polymer/lipid electrostatic interaction, steric forces from polymer chains extending into solution from the layer of polymer, vesicle osmotic stress, and surface roughness also contribute to the success of deposition and the stability of the bilayer [269].

It is evident from this review that a cushioned model membrane would be an excellent platform to pursue detailed studies of the role of the PL membrane in FGF1 translocation. The HBM utilized by Doyle *et al*. [171] was a good starting point and provided critical information regarding membrane transport, however, it was limited in that it was not a true PL bilayer, and did not facilitate protein transport. Rather, by employing a cushioned model membrane, with its inherent two PL leaflets and a suitable cushion material, the membrane would be far more physiologically relevant and provide the structure for potential protein translocation. In addition, there is a precedence for utilizing cushioned membranes in SFS studies and in z-scan FCS studies.

## 9. Conclusions

Nonclassical export of extracellular signal peptide-less proteins, including FGF1, involves their translocation through the PL bilayer. In the case of FGF1, this process involves the formation of a multiprotein release complex composed of several signal peptide-less PL-binding polypeptides and dependent on copper ions. Studies on cell-free systems including artificial PL bilayers demonstrated that proteins of FGF1 MRC induce membrane destabilization. The understanding of polypeptide translocation through the cell membrane requires further development of cell-free experimental models and methods of detection of membrane perturbation and transmembrane protein transport.

## Abbreviations

Anx 2: annexin 2
BLM: black lipid membrane
ER: endoplasmic reticulum
FCS: fluorescence correlation spectroscopy
FGF1: fibroblast growth factor 1
HBM: hybrid bilayer membrane
IL1: interleukin 1
ITC: isothermal titration calorimetry
MRC: multiprotein release complex
PC: phosphatidylcholine
PI: phosphatidylinositol
PG: phosphatidylglycerol
PL: phospholipid
PS: phosphatidylserine
SAM: self-assembled monolayers
SFG: sum frequency generation
SFS: sum frequency generation vibrational spectroscopy
SphK1: sphingosine kinase 1
Syt1: synaptotagmin
